# Health Risk Implications of Volatile Organic Compounds in Wildfire Smoke During the 2019 FIREX‐AQ Campaign and Beyond

**DOI:** 10.1029/2021GH000546

**Published:** 2022-08-01

**Authors:** Gabrielle N. Dickinson, Dylan D. Miller, Aakriti Bajracharya, William Bruchard, Timbre A. Durbin, John K. P. McGarry, Elijah P. Moser, Laurel A. Nuñez, Elias J. Pukkila, Phillip S. Scott, Parke J. Sutton, Nancy A. C. Johnston

**Affiliations:** ^1^ Physical, Life, Movement, and Sport Sciences Division Lewis‐Clark State College Lewiston ID USA

**Keywords:** wildfire, smoke, VOC, benzene, health risk, FIREX‐AQ

## Abstract

Fire Influence on Regional to Global Environments and Air Quality was a NOAA/NASA collaborative campaign conducted during the summer of 2019. The objectives included identifying and quantifying wildfire composition, smoke evolution, and climate and health impacts of wildfires and agricultural fires in the United States. Ground based mobile sampling via sorbent tubes occurred at the Nethker and Williams Flats fires (2019) and Chief Timothy and Whitetail Loop fires (2020) in Idaho and Washington. Air samples were analyzed through thermal desorption‐gas chromatography‐mass spectrometry for a variety of volatile organic compounds to elucidate both composition and health impacts. Benzene, toluene, ethylbenzene, xylenes, butenes, phenol, isoprene and pinenes were observed in the wildfire smoke, with benzene ranging from 0.04 to 25 ppbv. Health risk was assessed for each fire by determining sub‐chronic (wildfire event) and projected chronic inhalation risk exposure from benzene, a carcinogen, as well as other non‐carcinogenic compounds including toluene, ethylbenzene, xylenes, and hexane. The cancer risk of benzene from sub‐chronic exposure was 1 extra cancer per million people and ranged from 1 to 19 extra cancers per million people for the projected chronic scenarios, compared to a background level of 1 extra cancer per million people. The hazard index of non‐carcinogenic compounds was less than one for all scenarios and wildfires sampled, which was considered low risk for non‐cancer health events.

## Key Points

- Smoke from four active wildfires was sampled via sorbent tubes between 2019 and 2020 in Idaho and Washington
- Over 100 volatile organic compounds were analyzed via thermal desorption‐gas chromatography‐mass spectrometry
- Human health risk due to benzene was calculated up to 19 extra cancers per million people

## 1 Introduction

Wildfires in the Western United States have increased in frequency and size in the last 20 years (Abatzoglou & Williams, 2016; Dennison et al., 2014; Liu et al., 2017) and area burned is projected to increase 78% in the Pacific Northwest by 2050 (Spracklen et al., 2009). These changes in environmental behavior are attributed to anthropogenic climate change, as well as forest management methods including fire exclusion (Hanan et al., 2021; Spracklen et al., 2009; Steel et al., 2015; Williams et al., 2019).

The Northwest region of the United States experiences a consistent wildfire season, and Oregon and Idaho had strong wildfire activity in both 2020 and 2021 (InciWeb, 2021). The Pacific Northwest region (Idaho, Washington, Oregon) of the United States is comprised of a diverse array of landscape, such as: mountainous areas, coniferous forests, as well as wide, grassy plains. The Northwest accumulates a large amount of its moisture in the winter months due to snow packing (Holden et al., 2018). Overall increasing temperatures, less snowpack, early snow melt, and drought in the summer, are all factors in creating fuels that are very dry and susceptible to ignition (Halofsky et al., 2020; Holden et al., 2018; Westerling et al., 2006; Wimberly & Liu, 2014). This increased fuel load plays a large role in the number of large‐scale wildfires that occur within the region (Holden et al., 2018; Wimberly & Liu, 2014). Within the last decade the region has experienced large wildfires (>100,000 acres), that include but are not limited to: the Carlton Complex Fire in Washington (256,100 acres), Biscuit Fire (494,000+ acres) and Labor Day fires (971,900+ acres) in Oregon, as well as the Cascade Complex fires (316,000+ acres) in Idaho (Abatzoglou et al., 2021; Halofsky et al., 2020; Stevens‐Rumann et al., 2016). Together with California, the Western US has experienced high frequency of fires. Two of the largest wildfires in California history, August Complex (over 1,000,000 acres burned) and Dixie (over 960,000 acres burned) fires occurred within the last year and a half (CAL FIRE., 2021). These fires from the Western US created smoke that traveled long distances, even to the eastern US coast, affecting air quality far from the source (US EPA, 2020, 2021).

Emissions from wildfires contain particulate matter, as well as a large variety of gas‐phase chemical compounds such as volatile organic compounds (VOCs) and various biogenic and oxygenated volatile organic compounds (BVOCs and OVOCs) (Maleknia & Adams, 2008; Sekimoto et al., 2018; Wentworth et al., 2018). The variability of what compounds are emitted is dependent on the type of fuel burned, fire temperature, smoke age, and fuel moisture (Akagi et al., 2011; Briggs et al., 2016; Gilman et al., 2015; Jaffe et al., 2020; Prichard et al., 2019). Reactive nitrogen species such as NO_x_, HONO, NH_3_, and HNO_3_ are found in wildfire smoke (Chai et al., 2019; Jaffe et al., 2020; Simpson et al., 2011). Oxidation of VOCs via OH (in the presence of NO_x_) plays an important role in production of secondary organic aerosols as well as tropospheric ozone (Chai et al., 2019; Jaffe et al., 2020).

Contributions to wildfire and biomass burning emissions include BVOCs, BTEX (benzene, toluene, ethylbenzene, xylenes), and PM_2.5_ (Gilman et al., 2015; Liu et al., 2017; Schauer et al., 2001; Urbanski et al., 2008). BVOCs found in wildfires include terpenes such as isoprene and pinene (Maleknia et al., 2009). Exposure to PM_2.5_ has been known to increase the risk of airway inflammation, cardiovascular disease, decreased heart rate, and acute adverse cardiac events (Chen et al., 2021; Haikerwal et al., 2015; Weichenthal et al., 2017). Animal and limited human studies have been conducted determine risks associated with exposure to BTEX and hexane (US EPA, 2021). Benzene is classified as a carcinogen, increasing the risk of leukemia (US EPA, 2021). Toluene can cause adverse neurological effects, while the xylenes have the potential to cause impaired motor function upon inhalation (US EPA, 2021). Ethylbenzene can cause throat and eye irritation, as well as kidney, lung, and liver cancer (NCBI, 2021). Hexane was shown to cause neuropathy (US EPA, 2021). The above associated health risks are determined via animal studies consisting of acute, high exposure to the specific gas‐phase compound. Although the concentrations found in wildfire smoke emissions may not reach these levels, these effects cannot be dismissed in their application to humans in the long‐term due to the intensifying wildfire seasons, especially in the Western United States. The impact may be cumulative. These health effects also leave room for studies covering wildfire smoke exposure and the health risks posed to firefighters, of which high level exposures easily could occur. There have been recent studies estimating exposure of PM_2.5_ and risk of mortality and other health impacts, including exposure models and anticipation of more wildfire smoke with climate changes (Ford et al., 2018; Lassman et al., 2017; Liu et al., 2016, 2021; Neumann et al., 2021; O’Dell et al., 2021). O’Dell et al. (2020) estimate wildfire exposures for a few VOCs such as benzene based on aircraft measurement extrapolation, but overall, less has been reported in literature on gas phase health risks versus particulate matter.

Fire Influence on Regional to Global Environments and Air Quality (FIREX‐AQ) was a collaborative campaign led by the US National Oceanographic and Atmospheric Administration and National Aeronautics Space Administration (NOAA/NASA) carried out during the 2019 summer wildfire season. The main goals of the campaign were to study agricultural and wildfire smoke composition, as well as smoke plume evolution and diurnal changes in atmospheric chemistry to better understand the impact of fires on the climate and air quality (Warneke et al., 2018). The collaborative project consisted of a variety of measurements and products from over 50 agencies and universities, including Lewis‐Clark State College (LCSC) based in Lewiston, ID. Ground sampling results from the LCSC VOC Research Group were published under the NASA FIREX‐AQ archived database along with other participating institutions (NASA, 2020).

LCSC's contribution to this collaborative effort was analyzing the VOC composition of wildfires at ground level to determine human health risks of wildfire smoke. A large breadth of research has been done to characterize emissions of wildfires in a laboratory setting or via aerial measurements (Koss et al., 2018; NASA, 2020; Permar et al., 2021; Warneke et al., 2018). There have been multiple studies done on the composition of wildfire smoke, however, there is a lack of studies correlating these emissions to human health risk. There is also an abundant amount of discussion surrounding human health risk related to particulate matter (PM_2.5_) exposure from wildfires, but less so with VOCs. One recent study exploring health risks related to VOCs from wildfire smoke was calculated through extrapolation from concentrations found via aircraft sampling (O’Dell et al., 2020). The main objective of this study was to explore not only wildfire smoke composition, but also correlate findings to human health risk on the ground where normal exposure occurs. The concentrations found at the ground level more accurately represent air quality and the impact on the health of the residents and communities near wildfire influence and downwind.

## 2 Materials and Methods

### 2.1 Fire Smoke Sampling

**Figure 1:**
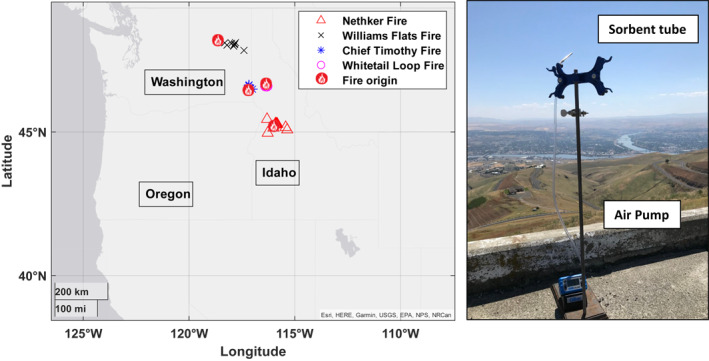
Left: Sampling locations in proximity to the origin of each fire (maps produced with MATLAB, 2019). Fire symbols are representatives of origin, various symbols are representative of samples taken. Right: Sampling apparatus consisting of sorbent tube and pump looking south along the Idaho/Washington state border.

Ambient air grab samples were collected at wildfires in various locations across Washington and Idaho. Wildfire samples were collected actively with Markes International dual sorbent thermal desorption tubes packed with Tenax®TA‐Sulficarb. Collection occurred with Gilian® GilAir® Plus and Markes ACTI‐VOC pumps at a rate of 200 mL/min with volume collected ranging from 0.5 to 2.0 L, and time durations of 2.5–10 min. Variability in volume collected (and thus, duration) depended on how heavy smoke presence was (over smolder or close to fire). Glass wool was placed at the sampling end of sorbent tube to filter particulate matter when heavy smoke was observed. Figure 1 shows sampling locations relative to the origin of the fire as well as the sample set up.

Active samples were taken at various locations on several dates, including in areas being affected by the Nethker (Central Idaho, 8, 11, 15, 30‐August; 1, 3, 4‐September) and Williams Flats (Eastern Washington, 3, 5‐August) fires in 2019, as well as the Chief Timothy in Southeastern Washington, 16, 17, 19‐August) and Whitetail Loop (North Central Idaho, 1‐September) fires in 2020. Samples taken ranged from 2.5 to 10 min each depending on severity of smoke. Duplicates and field blanks were taken to account for any variability or shipping contamination. Passive samples (7‐day averaging) taken at stationary sites served as background concentrations for compounds investigated for health risk (Miller et al., 2022). These diffusive samples collected ambient air for one week to one month during time periods of minimal to no wildfire influence in the following areas: McCall, Moscow, Lewiston, and Boise, ID, as well as Spokane, WA and Missoula, MT (Chandra et al., 2020; Miller et al., 2022; NASA, 2020). More in‐depth details on this background data set can be found in Miller et al. (2022). Details about fires sampled are found in Table 1.

**Table 1:** Overview of Wildfires Sampled (InciWeb, 2021)

Fire	Closest city	Origin of fire	Start date	Duration (days)	Cause	Fuels	Acres burned	Samples (n)	Miles from origin (range)
Nethker	McCall, ID	45.246, −115.93	4‐August 2019	31	Lightning strike	Douglas fir, Ponderosa pine	2,360	22	1–30
Williams Flats	Keller, WA	47.98, −118.624	2‐August 2019	24	Lightning strike	Douglas fir, Ponderosa pine, short grass, bitterbrush, slash, mixed conifer overstory	44,446	10	16–61
Chief Timothy	Clarkston, WA	46.426, −117.189	16‐August 2020	4	Human caused	Grass, brush	1,400	14	1–9
Whitetail Loop	Orofino, ID	46.513, −116.345	31‐August 2020	6	Unknown	Ponderosa pine, tall grass, brush	499	6	0–2

### 2.2 Analysis and Quality Control

Samples were returned to the laboratory, purged with ultra‐high purity nitrogen for 5 minutes to reduce water vapor, and then analyzed via thermal desorption‐gas chromatography‐mass spectrometry (TD‐GC‐MS), using a method adapted from EPA method TO‐17 (US EPA, 1999). After samplin Table 2 summarizes instrumental methods, and more detailed descriptions can be found in Scott et al. (2020) and Miller et al. (2022).

**Table 2:** Overview of Instrumental Specifications for Thermal Desorption‐Gas Chromatography‐Mass Spectrometry Analysis

*Thermal Desorption Unit:* Markes Unity 2	
Carrier Gas	He, 25 mL/min flow
Tube Desorption	Initial Temperature: 50°C for 5 min
	High Temperature: 200°C for 10 min
Cold Trap	Injection Time: 10 min
	Low Temperature: 30°C
	High Temperature: 250°C
Split Ratio	No Split → 1:1, Single → 1:2, Double→ 1:35

*Gas Chromatograph:* Agilent 7820 GC	
Carrier Gas	He, 1.5 mL/min flow
Mode	Constant flow
Column	Agilent DB‐624 UI, 60 m × 320 μm x 1.8 μm
Temperature Program	40°C for 2.0 min
	40°C to 195°C at 10°C/min with 12.5 min hold

*Mass Spectrometer:* Agilent 5977 MS	
Type	Quadrupole, Electron Ionization
Detection Mode	Scan and trace ion, m/z 45–300
Source Temperature	230°C
Quad Temperature	150°C

Standardization of 106 compounds was completed using both liquid and gas standards. Gas standardization for VOCs utilized an Airgas TO‐15/17 62 component VOC mix and a PAMS 57 component mix at 1 ppm in nitrogen. Liquid standardization of terpenes utilized 100 μg/mL SPEX CertiPrep® CAN‐TERP‐MIX2. Calibration of dimethyl sulfide and dimethyl disulfide (DMS and DMDS, Alfa Aesar and Acros Organics, respectively), as well as select alcohols (phenol, syringol from Acros Organics; guaiacol from Sigma‐Aldrich) was completed by preparing 100 ppmv liquid stock solutions with each pure compound in HPLC grade methanol.

Calibration levels for VOCs ranged from 0.1 to 10 nL, with 10 nL being the upper limit of detection (ULOD). Terpene and alcohol calibration levels ranged from 0.05 to 40 ng, with the ULOD at 40 ng. All units were converted to ppbv using raw amounts detected via analysis/calibration, volume sampled, the molecular weight and molar volume at 1 atm and 298 K. Calibrations were checked weekly for 80%–120% recovery, with recalibration occurring upon any compound with a quantification outside of this range. TD‐GC‐MS details including compound name, molecular weight, empirical formula, retention time, quantization ion, linearity, and limit of detection (LOD) for each compound are shown in Table S1 in Supporting Infomation S1. Detection limits were as low as 0.002 ppbv. Gas chromatograms were individually assessed for accuracy and then manually integrated, using Agilent MassHunter Enhanced Data Analysis. Peaks of interest were verified through a NIST mass spectral library and qualitative mass to charge ions (m/z). Duplicate pairs result in 5% precision.

### 2.3 Health‐Risk Assessment

To assess the health risk of compounds found in wildfire smoke, the upper confidence limit (UCL) of the measured data set for each air toxic compound and fire were calculated using United States Environmental Protection Agency's ProUCL 5.1 software (US EPA, 2016). The UCL is generated through a statistical analysis that translates to there being a probability that 95% of sample concentrations will lie below the UCL when fit with a normal or other distribution. The use of a UCL in risk assessment versus the mean is standard EPA practice to overestimate the risk rather than underestimate the risk. More in depth explanations of the software's function can be found in Singh and Maichle (2015). Sample concentrations were obtained from dual‐sorbent tube sampling as outlined above and statistical data were analyzed with EPA ProUCL 5.1 (US EPA, 2016). The wildfire samples were taken via active (pumped) sampling, while the data representative of the background was taken through passive (diffusive) sampling. The background sampling sites chosen to correlate to each fire were those into the closest proximity. The Nethker Fire background was established via a site in McCall, ID, Chief Timothy and Whitetail Loop via a site in Lewiston, ID, and Williams Flats via a site in Spokane, WA (Miller et al., 2022; NASA, 2020). ProUCL 5.1 (2016) software was also used to generate Kaplan‐Meier statistics using non‐detect values. Non‐detect values consisted of values that either were below the LOD or the compound was truly not detected by the system. Non‐zero values, those below the LOD, were inputted into the software, while zeroes were replaced with LOD/2. Concentrations over the ULOD were replaced with the respective values from the calibration curve's upper limit.

The UCL generated from the software was used as the contaminant concentration in air (CA) in the risk calculations. Several wildfire exposure scenarios were calculated: sub‐chronic (wildfire event) and chronic projections of repeated wildfire events (occupational, residential and lifetime). Sub‐chronic and chronic exposure and cancer risk (benzene only) were computed from these values. Acute exposure was not employed for risk analysis in this scenario because acute risk is defined by the EPA as 24 hr or less (US EPA, 2009). Sub‐chronic exposure is defined as repeated exposure for over 24 hr lasting up to 10% of the human lifespan of 70 years (US EPA, 2009). The hazard quotients for non‐carcinogenic compounds were also assessed. The inhalation unit risk (IUR) and reference concentration values (RfC) for both assessments were obtained through the EPA IRIS library (US EPA, 2021). The RfC of a compound is representative of chronic exposure of non‐carcinogenic nature which may result in an adverse health outcome; however, it is common practice by the EPA to use both the IUR and RfC for sub chronic and chronic exposure in carcinogenic and hazardous air pollutant (HAP) risk‐assessment.

Sub‐chronic to chronic exposure was calculated by using the following equation (US EPA, 2009):

(1) EC=∑(CA×ET×EF×ED)/AT

where CA is the contaminant concentration in air (μg/m^3^) is the UCL obtained from ProUCL 5.1 software (US EPA, 2016),

ET is the exposure time in hours per day, EF is the exposure frequency in days per year, ED is the exposure duration in years, AT, is the averaging time in hours, or 24 hr/day × 365 days/yr × 70 years.

The scenario types and values of the above variables used are summarized in Table 3. The “wildfire event” scenario was based on sub‐chronic one month exposure to the emissions observed in this study for a given compound and wildfire. The “occupational” scenario was based on EPA's designation of the amount of time someone spends at work (250 days), modified for seasonal (summer) wildfire fighters to be 90 days. The “residential” scenario was based on the EPA's designation of the average length a person lives in a single location, or 26 years (US EPA, 2009). The “lifetime” scenario was defined by the EPA's designation of the average human's life expectancy, or 70 years (US EPA, 2009). These values are used in the ED portion of the equation. The wildfire exposure was set at 30 days for each scenario, except the occupational, to reflect the exposure of one living near the wildfires. This time frame was chosen for uniformity of analysis and the assumption is made that exposure concentrations (ECs) would remain similar for the entire month's period. The chronic scenarios we used were repeated exposure for the occupational, residential and lifetime, meaning these fires would reoccur each year for 25, 26 and 70 years, respectively. Note, these are projections for risk only. These scenarios are summarized in Table 3 for both fire and background and calculations are based on standard protocols for health risk (US EPA, 2014). The result produces the EC, in μg/m^3^. See Miller et al. (2022) for detailed passive sampling data used in this assessment.

**Table 3:** Summary of Health Risk Scenarios Used to Calculate Exposure Concentrations (EC), Based on Ground Sampling Near Wildfires (Fire) and Background (Bg) Sites

Exposure Type	ET (Fire)	EF (Fire)	ED (Fire)	ET (Bg)	EF (Bg)	ED (Bg)
Wildfire event	24	30	1	24	320	26
Occupational^a^	8	90	25	16	275	25
Residential^a^	24	30	26	24	320	26
Lifetime^a^	24	30	70	24	335	70

*Note.* Values are based on guidance from US EPA (2014).

^a^ Projected chronic exposure.

This value (EC) was then used to calculate a cancer risk in the case of benzene, a known carcinogen:

(2) CancerRisk=IUR×EC

The calculated risk determines how many additional cancers per one million people could occur due to this exposure. For benzene, the IUR of 7.8 × 10^−6^ (μg/m^3^)^−1^ was used (US EPA, 2021). For a full example calculation with our data, please see Figure S1 in Supporting Information S1.

The potential for non‐cancerous adverse health effects was calculated through a hazard quotient (HQ). The hazard quotient was calculated through the following equation:

(3) HQ=EC/RfC

An HQ greater than one is indicative that the estimated exposure may cause adverse non‐carcinogenic health effects. If the HQ is less than one, then non‐carcinogenic health effects are not expected to occur. The hazard index (HI) is the sum of HQs for compounds both present in samples and having an available RfC. In this assessment, the HI included benzene, ethylbenzene, hexane, xylenes, and toluene for each wildfire.

## 3 Results and Discussion

### 3.1 Chemical Composition of Fires

**Figure 2:**
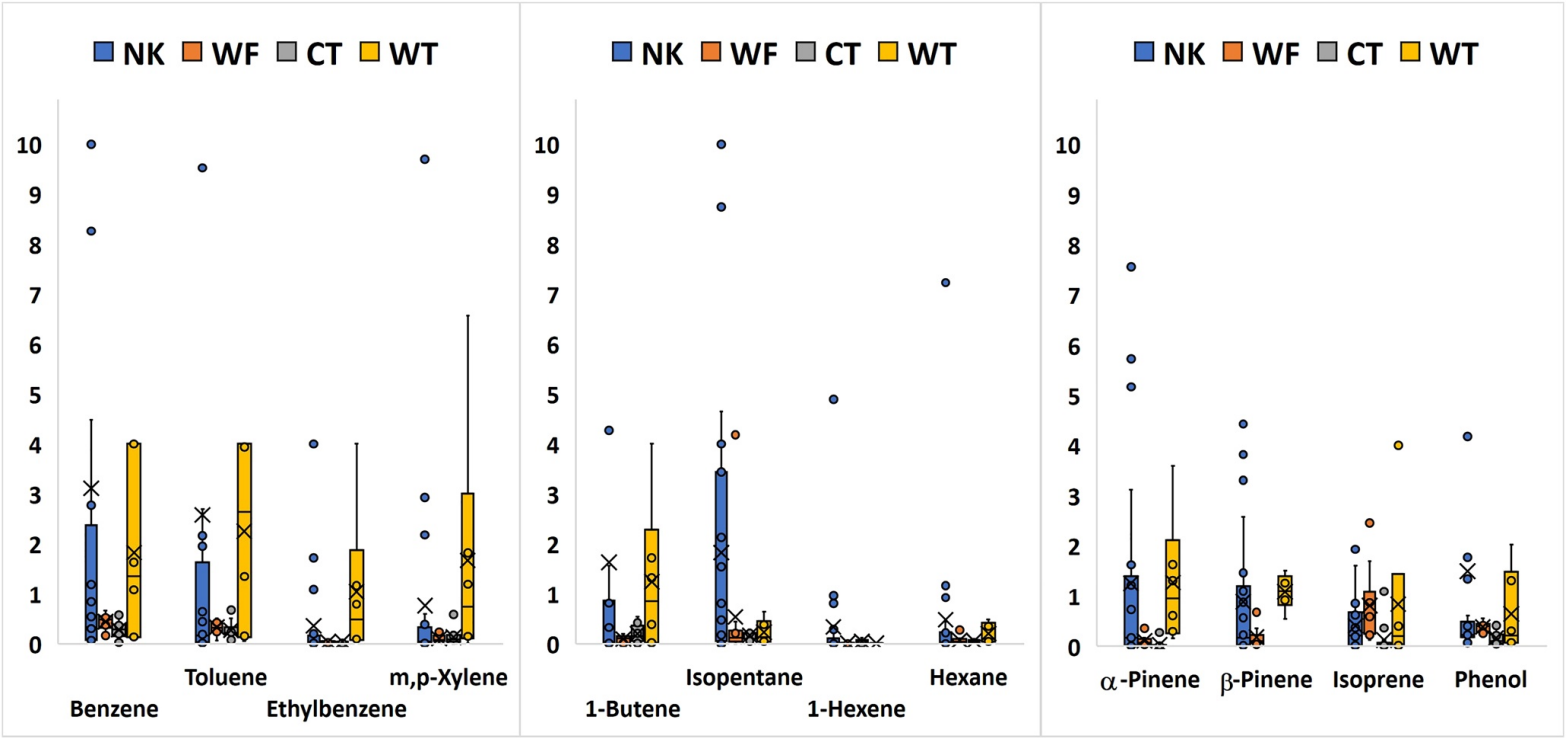
Box‐and‐whisker plots show the quartile distributions, mean (x) and median (−) for benzene, toluene, ethylbenzene, xylenes (a), select aliphatic hydrocarbons (b), and select biogenic/oxygenated compounds (c). All values are expressed in ppbv. Symbols for fires are Nethker (NK), Williams Flats (WF), Chief Timothy (CT), and Whitetail Loop (WT).

Table 4 shows general statistics from samples taken at the 2019 Nethker and Williams Flats fires, and Table 5 shows general statistics from samples taken at the Chief Timothy and Whitetail Loop fires of 2020. Statistics were generated from raw concentrations inputted into EPA's ProUCL 5.1 (US EPA, 2016), including non‐detects. As shown in Table 1, the Nethker, Williams Flats, and Whitetail fires were primarily fueled by fir and/or pine trees, while Chief Timothy was fueled by grass and brush. Whitetail Loop and Williams Flats also included grass as a fuel. Samples taken at all fires were in active visible smoke, with the Nethker fire in closest proximity to the smoldering fire, which is shown by the overall higher maximum values and averages compared to the others. Williams Flats sampling occurred the farthest from the fire, due to geographical obstacles, and thus, tended to have some of the smallest concentrations of VOCs, even though it was the largest fire of the four. Select VOCs are shown in box and whisker plots in Figure 2.

**Table 4:** Statistics for 2019 Fire Influence on Regional to Global Environments and Air Quality Fires Generated With ProUCL 5.1 Including Number of Samples (n), the Percent Samples Non‐Detected (% ND), Range, Mean ($\bar{x}$), and Standard Deviation (s) for Compounds Found in the Nethker and Williams Flats Fires, Respectively

Compound	Nethker fire (n = 22)			Williams flats fire (n = 10)		
	% ND^b^	Range (ppbv)	$\bar{x}$ +/− s (ppbv)	% ND	Range (ppbv)	$\bar{x}$ +/− s (ppbv)
*Aromatic*						
Benzene	0	0.042–25.000^a^	3.968 ± 6.287	0	0.165–0.668	0.446 ± 0.167
Benzene, 1‐ethyl‐4‐methyl	0	0.011–1.740	0.132 ± 0.374	30	0.011–0.030	0.015 ± 0.006
Benzene, (1‐methylethyl)	73	0.040–1.900	0.155 ± 0.406	100	ND	ND
Benzene, 1,2,3‐trimethyl	73	0.046–2.530	0.182 ± 0.532	100	ND	ND
Ethylbenzene	41	0.006–5.780	0.439 ± 1.262	0	0.010–0.085	0.042 ± 0.021
Toluene	0	0.017–25.000^a^	3.445 ± 6.352	0	0.067–0.515	0.331 ± 0.135
Xylene (m,p)	0	0.007–9.700	0.768 ± 2.129	0	0.010–0.275	0.107 ± 0.086
Xylene (o)	5	0.007–3.280	0.169 ± 0.678	10	0.007–0.093	0.039 ± 0.026
*Hydrocarbon*
Isopentane	9	0.007–10.000^a^	1.826 ± 2.798	10	0.007–4.180	0.541 ± 1.220
1‐Butene	50	0.027–25.000^a^	2.034 ± 5.507	60	0.027–0.164	0.096 ± 0.072
Pentane, 2‐methyl	95	ND	ND	70	0.040–0.417	0.081 ± 0.122
Pentane, 3‐methyl	95	ND	ND	70	0.035–0.258	0.059 ± 0.078
2‐Pentene (Z), cis	68	0.015–5.200	0.332 ± 1.091	70	0.015–0.144	0.025 ± 0.041
Hexane	36	0.007–7.230	0.481 ± 1.503	0	0.013–0.280	0.074 ± 0.079
Hexane, 2‐methyl	95	ND	ND	70	0.031–0.194	0.041 ± 0.059
Hexane, 3‐methyl	91	ND	ND	70	0.035–0.299	0.066 ± 0.094
1‐Hexene	50	0.011–4.890	0.338 ± 1.205	50	0.011–0.028	0.014 ± 0.009
Heptane	0	0.006–0.796	0.082 ± 0.176	50	0.006–0.081	0.031 ± 0.028
Octane	55	0.030–2.150	0.169 ± 0.447	100	ND	ND
*Oxygenated*
2‐Hexanone	68	0.006–0.160	0.019 ± 0.036	70	0.006–0.015	0.006 ± 0.004
Guaiacol	41	0.011–14.772^a^	1.433 ± 3.273	100	ND	ND
Phenol	14	0.038–21.114	0.554 ± 0.910	0	0.193–0.551	0.382 ± 0.116
*Biogenic*
Camphor	64	0.016–0.915	0.091 ± 0.196	100	ND	ND
Isoprene	41	0.021–1.930	0.406 ± 0.527	0	0.124–2.460	0.808 ± 0.738
D‐Limonene	55	0.006–8.135	1.086 ± 2.154	90	ND	ND
α‐Pinene	32	0.016–7.561	1.250 ± 2.116	0	0.029–0.359	0.107 ± 0.100
β‐Pinene	23	0.033–4.433	0.886 ± 1.334	0	0.033–0.68	0.178 ± 0.200
Sabinene	64	0.019–1.511	0.185 ± 0.370	100	ND	ND
γ‐Terpinene	73	0.029–0.580	0.055 ± 0.121	100	ND	ND
*Sulfur*
Dimethyl sulfide	ND	ND	ND	70	0.014–0.060	0.017 ± 0.018

*Note.* Full data set can be found on NASA FIREX‐AQ archive (2020).

^a^ Denotes upper limit of detection (ULOD) value substituted.

^b^ Compounds not detected in more than 80% of samples are indicated as ND.

**Table 5:** Statistics for 2020 Fires Observations Generated With ProUCL 5.1 (US EPA, 2014) Including Number of Samples (n), the Percent Samples Non‐Detected (% ND), Range, Mean ($\bar{x}$), and Standard Deviation (*s*) for Compounds Found in the Chief Timothy and Whitetail Loop Fires, Respectively

Compound	Chief timothy fire (n = 14)			Whitetail loop fire (n = 6)		
	% ND^*b*^	Range (ppbv)	$\bar{x}$ +/− s (ppbv)	% ND	Range (ppbv)	$\bar{x}$ +/− s (ppbv)
*Aromatic*						
Benzene	0	0.024–0.596	0.283 ± 0.181	0	0.104–4.000^a^	1.826 ± 1.781
Benzene, 1‐ethyl‐2‐methyl	93	ND	ND	67	0.053–0.174	0.068 ± 0.060
Benzene, 1‐ethyl‐3‐methyl	71	0.063–0.248	0.042 ± 0.065	67	0.063–0.349	0.124 ± 0.133
Benzene, 1‐ethyl‐4‐methyl	50	0.011–0.107	0.021 ± 0.028	50	0.011–0.248	0.080 ± 0.099
Benzene, (1‐methylethyl)	100	ND	ND	67	0.040–0.204	0.074 ± 0.077
Benzene, 1,2,3‐trimethyl	71	0.012–0.117	0.024 ± 0.032	67	0.046–0.488	0.157 ± 0.193
Benzene, 1,2,4‐trimethyl	79	0.012–0.729	0.115 ± 0.220	50	0.012–0.850	0.292 ± 0.346
Ethylbenzene	7	0.006–0.138	0.042 ± 0.032	0	0.009–4.000^a^	1.042 ± 1.518
Mesitylene	50	0.013–0.127	0.026 ± 0.032	100	ND	ND
Styrene	43	0.007–0.147	0.031 ± 0.039	50	0.007–0.838	0.271 ± 0.333
Toluene	0	0.054–0.677	0.273 ± 0.165	0	0.059–4.000^a^	3.223 ± 3.541
Xylene (m,p)	7	0.007–7.140	0.132 ± 0.144	0	0.009–6.566	2.250 ± 1.947
Xylene (o)	29	0.007–0.227	0.043 ± 0.056	17	0.007–0.613	0.258 ± 0.225
*Hydrocarbon*						
Isopentane	0	0.043–0.286	0.174 ± 0.074	0	0.034–0.648	0.232 ± 0.240
1‐Butene	14	0.027–0.543	0.028 ± 0.167	33	0.027–4.000^a^	1.856 ± 1.534
2‐Butene (E), trans	93	ND	ND	50	0.008–0.860	0.228 ± 0.309
2‐Butene (Z), cis	93	ND	ND	50	0.009–1.404	0.351 ± 0.499
1‐Pentene	100	ND	ND	67	0.006–0.495	0.140 ± 0.200
2‐Pentene (Z), cis	64	0.015–0.050	0.016 ± 0.014	67	0.015–0.940	0.166 ± 0.346
Hexane	0	0.015–0.100	0.055 ± 0.027	0	0.010–0.485	0.171 ± 0.198
1‐Hexene	21	0.011–0.120	0.047 ± 0.035	100	ND	ND
Heptane	0	0.020–0.079	0.042 ± 0.020	83	ND	ND
Heptane, 3‐methyl	79	0.033–0.093	0.026 ± 0.021	100	ND	ND
Octane	57	0.030–0.090	0.038 ± 0.025	67	0.030–0.229	0.075 ± 0.094
Undecane	100	ND	ND	67	0.078–0.268	0.079 ± 0.104
Dodecane	100	ND	ND	67	0.055–0.146	0.059 ± 0.048
*Oxygenated*						
Borneol	100	ND	ND	50	0.022–3.17^a^	1.782 ± 1.229
Fenchol	100	ND	ND	67	0.021–0.115	0.038 ± 0.041
L‐Fenchone	93	ND	ND	67	0.018–0.085	0.030 ± 0.030
2‐Hexanone	36	0.006–0.020	0.011 ± 0.007	50	0.006–0.070	0.026 ± 0.028
Methyl Isobutyl Ketone	64	0.005–0.032	0.009 ± 0.010	100	ND	ND
Phenol	50	0.071–0.366	0.123 ± 0.108	33	0.071–1.533	0.485 ± 0.575
α−Terpineol	100	ND	ND	67	0.017–1.454	0.275 ± 0.530
Terpinolene	100	ND	ND	67	0.023–0.126	0.038 ± 0.043
*Biogenic*						
Camphor	93	ND	ND	50	0.016–3.212^a^	2.428 ± 1.568
Isoprene	71	0.021–1.090	0.124 ± 0.283	50	0.021–4.000^a^	1.660 ± 2.029
D‐Limonene	93	ND	ND	0	0.034–3.589^a^	1.266 ± 1.317
α‐Pinene	64	0.016–0.273	0.032 ± 0.067	0	0.162–3.590^a^	1.264 ± 1.274
β‐Pinene	86	ND	ND	0	0.541–1.505	1.083 ± 0.353
Sabinene	93	ND	ND	50	0.029–5.196^a^	1.954 ± 2.819
*Sulfur*						
Dimethyl Sulfide	71	0.014–0.188	0.041 ± 0.058	100	ND	ND

*Note.* Full data set can be found on Mendeley data (Johnston, 2021).

^a^ Denotes upper limit of detection (ULOD) value substituted.

^b^ Compounds not detected in greater than 80% of samples were omitted from this table and indicated as ND.

#### 3.1.1 The Nethker Fire (FIREX‐AQ)

The Nethker fire was sampled on several occasions (8, 11, 15, 30‐August and 1, 3, 4 of September 2019), with sampling conditions ranging from general low lying smoke to smoldering fires. BTEX compounds (from highest to lowest: benzene, toluene, xylenes, and ethylbenzene) were found to have elevated concentrations in the Nethker fire (Table 4). Benzene concentrations found were on average over 100 times higher than background, and similar trends of elevation were seen for toluene, ethylbenzene, and xylenes. For example, the average background concentration of benzene found in nearby McCall, ID was 0.044 ppbv during passive sampling taken during FIREX‐AQ (Miller et al., 2022). Benzene ranged from the background to 25 ppbv (the ULOD) with a mean of about 4 ppbv. Other compounds found above 1 ppbv were isopentane, 1‐butene, guaiacol, plus BVOCs limonene and alpha‐pinene (Table 4).

The Aerodyne mobile laboratory also took benzene and other VOC measurements during the Nethker fire as part of the FIREX‐AQ campaign but with a proton transfer mass spectrometer (NASA, 2020). Comparatively, their benzene values ranged from 0.04 to 73.66 ppbv with a median of 1.48 ppbv and mean of 6.51 ± 8.91 ppbv (NASA, 2020). The range and mean values are higher than what we observed, but considering the standard deviations, the means are comparable. The Aerodyne mobile laboratory spent more time in active fire plumes with higher time resolution sampling (averaged to 1 min intervals) (NASA, 2020).

#### 3.1.2 The Williams Flats Fire (FIREX‐AQ)

The Williams Flats Fire was primarily composed of timber and grass (Table 1). Sampling took place toward the beginning of the fire, on 3 and 5 August, 2019. Winds generally were from the north‐northwest and a large, wide fire plume was observed at the sampling sites east of the fire (Figure S2 in Supporting Information S1). Similar trends of elevation were seen for the BTEX concentrations but were almost a magnitude of 10 smaller compared to the Nethker Fire. Benzene averaged 0.4 ppbv and other concentrations decreased in the order, xylenes, toluene and ethylbenzene (all with means below 0.2 ppbv). Aliphatic HCs were detected but not relatively high, and included methyl pentanes and hexanes and 1‐butene. Isopentane was the most abundant of this group, with a mean over 0.5 ppbv. BVOCs were lower than 0.2 ppbv or non‐detects more than 80% of the time, except isoprene, which was 0.8 ppbv on average. Phenol was present in all the Williams Flats samples with a mean of about 0.4 ppbv.

The NASA DC‐8 aircraft also measured VOCs in plumes from Williams Flats fire during the FIREX‐AQ campaign, including the University of California Irvine's whole air sampling (UCI WAS) with GC/GC‐MS analysis. UCI samples were taken between 8,000 and 10,000 feet at altitude, with sample duration from about 30 to 60 s. BTEX was coincidently measured but at altitude on 3‐August 2019. Average UCI WAS benzene values were above 2 ppbv, toluene about 1 ppbv, and xylenes and ethylbenzene under 0.2 ppbv (NASA, 2020). These values were significantly higher than our ground measurements, suggesting the smoke plume remained high aloft. This high fire plume was observed while sampling, but smoke was still present on the ground (Figure S2 in Supporting Information S1).

#### 3.1.3 The Chief Timothy Fire (2020)

The Chief Timothy fire was exclusively fueled by grass and brush (InciWeb, 2021). Sampling occurred on 16, 17, 19‐August 2020, and in general, Chief Timothy fire samples had lower values of most VOCs observed compared to the other fires. Winds were variable and inconsistent (Johnston, 2021). Benzene and toluene had similar averages of about 0.3 ppbv with lower values of xylenes followed by ethylbenzene of the BTEX group (Table 5). Isopentane was the highest aliphatic HC detected with a mean of just under 0.2 ppbv, while phenol was one of few oxygenated compounds present. There is almost a complete lack of BVOCs in Chief Timothy fire samples, except α‐pinene and isoprene which had relatively low means of 0.03 and 0.1 ppbv, respectively. Dimethyl sulfuide (DMS) was detected in a few of these samples at low levels (mean 0.04 ppbv) but the fire was close to a pulp paper mill located in Lewiston, ID, which is known to emit DMS and might account for the low levels of DMS detected (Scott et al., 2020).

The fire was a few miles west of the Lewis‐Clark Valley, an area the authors have studied in detail previously (Scott et al., 2020). Benzene was measured during smoke events in 2017 and 2018 in this region, with averages of 0.55 ± 0.61 ppbv and highs over 3.5 ppbv. During these episodes, smoke blew in from other regions and the fires were non‐adjacent.

#### 3.1.4 The Whitetail Loop Fire (2020)

The Whitetail Loop fire was fueled mainly by Ponderosa pine and grass (Table 1). Sampling took place on 1‐September 2020 at altitudes below and to the south of the fire. Winds in general were from the north to west‐northwest (Johnston, 2021). BTEX were found to be elevated over 1 ppbv on average in Whitetail Loop fire, as was 1‐butene. Toluene was the highest elevated BTEX, followed by xylenes, benzene and ethylbenzene (Table 5). Other elevated compounds in Whitetail Loop were OVOCs and BVOCs. The largest BVOCs concentrations found were camphor, isoprene and sabinene (means of about 2 ppbv), d‐limonene and α‐pinene both at about 1 ppbv. The Whitetail Loop fire had many OVOCs detected, including borneol at almost 2 ppbv mean, fenchol, l‐fenchone, α‐terpineol, and terpinolene which were not found in other fires. Several aliphatic hydrocarbons were detected including butenes, pentanes, hexane, octane, undecane, and dodecane.

### 3.2 Fire Inter‐Comparisons and Correlations

Figure 2 shows the quartile distribution (box and whisker plots, with data points) of concentrations of select compounds, expressed in ppbv, found in the Nethker (NK), Williams Flats (WF), Chief Timothy (CT), and Whitetail Loop (WT) fires. Benzene concentrations were typically found to be under 4 ppbv for all the fires, with Nethker having outlier values as high as 25 ppbv (outliers above 10 ppbv not shown) (Figure 2). Concentrations of benzene found in Whitetail Loop had a larger spread and smaller mean than Nethker. Both Williams Flats and Chief Timothy fires have much lower BTEX than the former fires. On average, benzene was greater than toluene in the fires, with a benzene/toluene (B/T) ratio over 1 for Nethker and Chief Timothy and over 3 for Williams Flats, but was less than one (0.57) for Whitetail Loop. B/T ratios in wildfires should be greater than those of other emissions such as traffic (Zhang et al., 2016). For example, Austin et al. (2001) saw ratios of about 3 during laboratory burning, and emission factors reported by Koss et al. (2018) show B/T values of 1.5. The B/T ratio will increase as toluene reacts with species like OH more quickly than benzene. Thus, as smoke ages, the B/T ratio should go up. This fits with what we observed for Williams Flats fire, as B/T was the greatest at 3 and our samples were farthest from the main fire source during this event. Whitetail Loop had the smallest B/T ratio of the four fires, and the largest amounts of BTEX as a whole, as fresh smoke was sampled.

Figure 2b shows select aliphatic hydrocarbons. These compounds were chosen due to being the most frequently detected across all four fires. Isopentane had a larger spread for Nethker, with values spreading over 4 ppbv. The remaining three fires all have values less than 1 ppbv, small in comparison. Whitetail Loop has the largest amounts of 1‐butene, with values primarily over 4 ppbv. Hexane and 1‐hexene do not have a large spread for any of the fires. The exceptions are the few outliers in Nethker. The Whitetail Loop fire also had more butenes and pentenes present than the other fires (Tables 3 and 4). Generally, more aliphatic hydrocarbons were detected in the Williams Flats fire, including methyl pentanes and hexanes, which were not detected in the others. This reason is unknown, but could be due to other sources. The observation of isopentane is usually expected with biomass burning (Rossabi & Helmig, 2018), but rather, industrial sources, although this does not seem to be the case in the current study). Aliphatic VOCs are typically associated with oil and gas sources, but some can be seen in biomass burning as documented by experimental fires (Koss et al., 2018).

Figure 2c shows the most frequently detected BVOCs/OVOCs across all four fires. Nethker and Whitetail Loop both have the largest spread of concentrations of α‐pinene with the largest outliers being over 7 ppbv. Phenol and isoprene were highest on average in the Whitetail Loop fire. Isoprene was the most consistent of the BVOCs in the fires, with exception of Chief Timothy which was low in all BVOCs. The BVOCs are most abundant in fires with timber as fuel, whereas Chief Timothy had the least, with grass as fuel. Pinenes and isoprene are monoterpenes and typically associated with coniferous forests (Sumitomo et al., 2015). These can react with ozone formed in fires and also the hydroxyl radical, with lifetimes on the order of hours. Given their high reactivity, an aged fire may not have as many BVOCs compared to BTEX and we do see this general trend.

Sulfides were only seen in William Flats and Chief Timothy fires, in the form of DMS, not dimethyl disulfide. Williams Flats fire had sometimes detectable, but low levels of dimethyl sulfide (DMS) with a mean less than 0.02 ppbv, while the mean of the Chief Timothy fire was double that with some possibly originating from a local paper plant. Some studies have shown that DMS is not only emitted from grass and bushfire, but also from forest fires (Meinardi et al., 2003; Vettikkat et al., 2020). However, the current study does not support this consistently.

Across all fires and compounds, there is much variation with a lot of outliers due to the nature of the fire behavior and distance from the fire. To further investigate relationships, the concentrations of compounds observed in the fires were analyzed through correlational analysis and the results of paired relationships are shown in Table 6. Benzene and toluene were correlated in all fires except Williams Flats, which may be due to the longer distances from this fire site. Ethylbenzene and toluene were correlated in all fires, but slightly less so in Whitetail Loop. It makes sense that BTEX are correlated and because of the elevation in the samples, can be attributed to the smoke source. Benzene concentrations strongly correlated to phenol concentrations (an oxidative form of benzene) in the Whitetail Loop and Nethker fires but not the others. Nethker had correlations with benzene and a‐pinene as well. Alpha‐pinene and isoprene were correlated in both Williams Flats and Whitetail Loop. The Nethker fire also had associations with ethylbenzene and phenol as well as octane and 1‐hexene. The only other fires with octane correlations to ethylbenzene was Chief Timothy, which also had a strong association with octane and heptane. Chief Timothy was the fire closest to industry, as it was near a shipping port along Snake River. In this fire it's possible to have some industrial influence as high correlations with ethylbenzene and toluene as well as octane with ethylbenzene and heptane. The two fires that had grass fuel (Williams Flats and Chief Timothy) were expected to show similar correlations, which was not confirmed with the exception of ethylbenzene/toluene. The timber fires (Nethker, Williams Flats, Whitetail Loop) also did not exhibit similar correlations, suggesting each fire had its own signature of gas emissions. These VOC composition results can be of value in comparing future studies, but the remaining discussion will focus on BTEX, especially benzene as the main carcinogen measured, and a few other compounds that may play a role in the health risk.

**Table 6:** Coefficient of Determination (R^2^) Values Resulting From Regression Analysis Between Compounds Found in Each Fire, With Values Over 0.8 Bolded

	Nethker	Williams Flats	Chief Timothy	Whitetail
				Loop
Benzene/α‐Pinene	0.79	0.09	−0.11	0.24
Benzene/Isopentane	0.07	−0.57	0.71	**0.87**
Benzene/Phenol	**0.88**	0.19	−0.33	**0.91**
Benzene/Toluene	**0.97**	0.31	**0.89**	**0.87**
Ethylbenzene/1‐Hexene	**0.99**	0.50	0.78	ND
Ethylbenzene/Octane	**0.98**	ND	**0.85**	−0.02
Ethylbenzene/Phenol	**0.99**	−0.53	−0.50	−0.06
Ethylbenzene/Toluene	**0.97**	**0.92**	**0.98**	0.67
Xylene (m,p)/Hexane	**0.98**	0.68	0.71	0.09
Octane/Heptane	0.11	ND	**0.95**	ND
Isoprene/α‐Pinene	−0.22	**0.82**	0.30	**0.92**
Phenol/D‐Limonene	0.75	ND	ND	0.12

*Note.* ND is indicative of that compound having more than 80% non‐detects for that fire and thus was excluded from the correlation analysis. Pairs containing a compound non‐detected in more than 80% of samples are indicated (ND).

### 3.3 Spatial Distribution of Benzene in Fires

**Figure 3:**
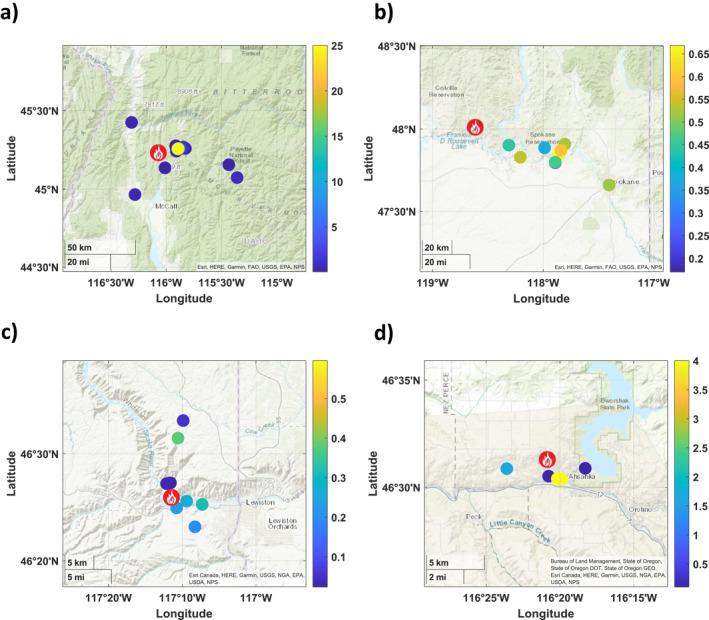
Benzene concentration maps (in ppbv) at samples taken during fires: (a) Nethker (b) Williams Flats (c) Chief Timothy (d) Whitetail Loop fire (maps produced with MATLAB, 2019).

The benzene concentrations measured at each sampling location for each fire are shown in Figure 3. The flame symbol is indicative of the origin of the fire, with color scale in ppbv units of benzene ranging from low (blue) to high (yellow). Note the numerical scales vary for each fire. Figure 3a shows Nethker fire near McCall, Idaho, with very large concentrations at the site of the active fire, and low concentrations around or at distance from it. There was a wide range of values, up to 25 ppbv, due to sampling of active smolders. Figure 3b shows sampling for the Williams Flats fire. Benzene concentrations were variable to the east of the fire, and often sampling caught the downwind plume (traveling east), but sometimes was on the southern edge of the smoke (Figures 3 and S2 in Supporting Information S1). Winds generally were north‐northwesterly on 3 August and westerly on 5 August at the site. Smoke influence was seen as far as Spokane, as benzene levels were elevated to 0.5 ppbv.

Figure 3c shows the concentration of benzene found for each sample taken at the Chief Timothy fire. Based on the origin of the fire, the samples taken across the river from the fire were in the path of the wind that was carrying smoke. These green markers show higher elevation of benzene (0.3–0.4 ppbv) versus the dark blue markers north of the origin (0.2 ppbv and below). The highest concentration of benzene near 0.4 ppbv was likely from settling of the smoke in the Snake River valley, as well as in the canyons as shown in the light green at second most northernly site.

Finally, Figure 3d shows the concentration of benzene found in each sample at the Whitetail Loop fire. Like Chief Timothy results, the sample taken the closest did not contain the highest concentration of benzene. This was likely due to the smoke plume emitted vertically, and then spreading outward with northwestern winds. The two samples on the outskirts of the maps have low concentrations. The markers in yellow were the highest concentrations of benzene found and north to northwest winds transported smoke toward these sites (Johnston, 2021). Benzene was highly variable in the fire smoke sampled from these four fires and ranged from 0.02 to over 25 ppbv. Some comparisons mentioned earlier were Aerodyne mobile laboratory data concentrations of benzene ranging 0.04–73 ppbv in the Nethker fire (NASA, 2020). UCI WAS measured in Williams Flats ranged from 0.02 to 8 ppbv (NASA, 2020). In the CAMP fire in California, benzene was measured at a maximum of 1.5 ppbv (Simms et al., 2021). The variability between these results can be due to many causes, especially the proximity of the sampling to the fire. The Simms et al. (2021) benzene concentration results were from a residential area. UCI WAS samples were from aerial plumes collected via aircraft. Our best agreement was with Aerodyne measurements that were often collocated and ground samples. Some researchers have shown that variability in VOC emission factors, including benzene, are highly correlated to modified combustion efficiency (MCE) of the fire (Permar et al., 2021). Even though there is variability in benzene levels measured at ground sites near wildfires, these are better to use than other estimates of VOCs or models when it comes to health risk, as these are the concentrations inhaled by persons living near wildfires. In all fires, there were homes and communities nearby.

### 3.4 Health‐Risk Assessment Results

VOCs emitted from wildfires pose potential health risks for those in communities that experience a consistent fire season and possibly even communities downwind from wildfires, as well. This risk is in part due to the carcinogenic nature of certain compounds that can be emitted, such as benzene (Gilman et al., 2015). A health‐risk analysis was performed to determine the risk for the actual wildfire events (30 days), and then projected scenarios of chronic, repeated exposures for occupational (25 years), residential (26 years) and lifetime (70 years) risk. In all cases (Johnston, 2022), the concentrations found from inhalation of gaseous compounds emitted from wildfire smoke measured at ground sites in 2019–2020. This risk estimates how many extra cancers per one million people could potentially occur if the exposure to benzene levels occurred one month out of every year for each scenario. A risk of one extra cancer per million people is considered low/background risk (US EPA, 2009).

Table 7 shows the UCL of benzene in μg/m^3^ calculated for each fire using LCSC's observed data and ProUCL 5.1 (US EPA, 2016). The wildfire event scenarios at all four wildfires resulted in low cancer risk due to benzene, or 1 × 10^−6^ (one extra cancer per million people). Although the benzene levels were high, the 30 days exposure is diluted over a lifetime of background exposure. Elevated cancer risk was seen with the chronic scenarios for the Nethker and Whitetail Loop fires, while Williams Flats and Chief Timothy were not significantly increased. The residential scenario was elevated above background levels for both Nethker and Whitetail Loop, with 7 and three extra cancers per million people; however, these values are still considered relatively low risk. Williams Flats and Chief Timothy fires were found to be background levels of risk. The risk for the occupational scenario was about the same as the residential scenario.

**Table 7:** Cancer Risk and Non‐Cancer Risk Analysis for Select Compounds for Each Fire Sampled

Compound	Fire	IUR^a^	CA (Fire)	CA (Bg)	Cancer risk (Event)	Cancer risk (Occ)	Cancer risk (Res)	Cancer risk (Life)
Benzene	NK	7.8E−06	26.39	0.26	1E−06	6E−06	7E−06	19E−06
	WF	7.8E−06	1.73	0.30	1E−06	1E−06	1E−06	3E−06
	CT	7.8E−06	1.18	0.27	1E−06	1E−06	1E−06	3E−06
	WL	7.8E−06	10.51	0.27	1E−06	3E−06	3E−06	9E−06

Compound	Fire	RfC^a^	CA (Fire)	CA (Bg)	HQ (Event)	HQ (Occ)	HQ (Res)	HQ (Life)
Benzene	NK	3E+01	26.39	0.26	4E−03	3E−02	3E−02	8E−02
	WF	3E+01	1.73	0.30	4E−03	3E−03	5E−03	1E−02
	CT	3E+01	1.18	0.27	3E−03	3E−03	4E−03	1E−02
	WL	3E+01	10.51	0.27	4E−03	1E−02	1E−02	4E−02
Ethylbenzene	NK	1E+03	7.00	0.02	2E−05	2E−04	2E−04	6E−04
	WF	1E+03	0.23	0.15	5E−05	3E−05	6E−05	2E−04
	CT	1E+03	0.29	0.11	4E−05	3E−05	5E−05	1E−04
	WL	1E+03	9.95	0.11	5E−05	3E−04	3E−04	9E−04
Hexane	NK	7E+02	6.81	0.07	5E−05	3E−04	3E−04	9E−04
	WF	7E+02	0.51	0.16	8E−05	6E−05	1E−04	3E−04
	CT	7E+02	0.24	0.18	9E−05	5E−05	9E−05	3E−04
	WL	7E+02	1.17	0.18	9E−05	9E−05	1E−04	4E−04
Toluene	NK	5E+03	11.49	0.25	2E−05	2E−04	2E−04	5E−04
	WF	5E+03	1.54	1.30	9E−05	6E−05	9E−05	3E−04
	CT	5E+03	1.32	0.56	4E−05	3E−05	4E−05	1E−04
	WL	5E+03	14.52	0.56	4E−05	1E−04	1E−04	3E−04
Xylene (m,p)	NK	1E+02	15.64	0.13	6E−04	5E−03	5E−03	1E−02
	WF	1E+02	0.68	0.80	3E−03	2E−03	3E−03	8E−03
	CT	1E+02	0.88	0.63	2E−03	1E−03	2E−03	6E−03
	WL	1E+02	16.17	0.63	2E−03	6E−03	7E−03	2E−02
Xylene (o)	NK	1E+02	3.54	0.02	1E−04	1E−03	1E−03	3E−03
	WF	1E+02	0.24	0.07	3E−04	2E−04	3E−04	9E−04
	CT	1E+02	0.44	0.08	3E−04	3E−04	4E−04	1E−03
	WL	1E+02	2.02	0.08	3E−04	7E−04	9E−04	2E−03
Hazard Index	NK				5E−03	3E−02	4E−02	1E−01
	WF				7E−03	5E−03	8E−03	2E−02
	CT				6E−03	5E−03	7E−03	2E−02
	WL				7E−03	2E−02	2E−02	6E−02

*Note.* CA (fire and background, bg), and RfC units are μg/m^3^, IUR units are (μg/m^3^)^−1^, HQ, HI are unitless, and cancer risk is number of extra cancers per million people.

^a^ US EPA, 2021.

For the lifetime cancer risk assessment, all four fires had a risk that was above background. The concentrations found in the Nethker and Whitetail Loop fires had the potential to cause 19 and nine extra cancers per million people, respectively, while Williams Flats and Chief Timothy had a risk of three extra cancers per million people. This is comparable to a risk assessment done by O’Dell et al. (2020) that ranges 1–25 × 10^−6^ for smoke exposure in the Northwestern US which used airborne measurements to model multiple carcinogenic compounds, including benzene, on the ground. O’Dell et al. (2020) also calculated health risk for compounds of interest, such as: acrylonitrile, formaldehyde, and acetaldehyde. A limitation of this study is that not every wildfire emission relevant compound is measured, however, one advantage of our analysis is the use of ground level measurements and concentrations and not extrapolations or models.

Risk calculations were also performed for non‐carcinogenic compounds. The metric for non‐cancer risk assessment is any hazard quotient calculated resulting in a value over one indicates the potential for an adverse health event to occur. The HI is the sum of the hazard quotients, and for all fires, this was below 1 for the compounds investigated, representing a low risk for non‐cancer events. Nethker and Whitetail Loop fires had the higher cancer risk for benzene of the four studied. They both had timbre as the main fuel source and had higher levels of most VOCs comparatively. These fires also had the highest non‐cancer risk or hazard indices values for the sum of compounds. It should be noted that the occupational, residential and lifetime scenarios were chosen to represent repeated exposure to fires of this nature.

### 3.5 Uncertainties and Limitations

Some limitations must be noted. Not all compounds measured were air toxics with existing reference concentrations (IUR or RfC) to calculate health risk, nor were all toxic compounds measured. Compounds that are found in biomass burning that were not measured in this study include, but are not limited to: bromoform, acrolein, furan, carbon monoxide, and hydrogen cyanide (O’Dell et al., 2020; Simpson et al., 2011). A gap in the data that would be helpful for a more well‐rounded picture of biomass burning emissions amongst different sources would be the addition of organic acids and furans. Something that was not measured in this study were VOC concentrations correlated to particulate matter (PM_2.5_) data. Elevated PM_2.5_ is a good indicator of wildfire influence; however, the focus of this manuscript was VOC speciation and how those VOCs might cause adverse health events. We do not measure carbon monoxide with our method, and thus we do not calculate emission factors for the compounds measured. This is not important in the assessment of risk, as the concentrations are what are directly used in risk assessment.

This specific inhalation risk assessment does not factor in if there are other risks of cancer and non‐cancer events in locations near wildfire influence, such as: heredity, lifestyle, occupational hazards, etc. Cancer can develop from exposure to other factors contributing at the locations measured, however, these calculations are specifically for determining risk of exposure to that individual compound. Furthermore, the risk is not predictive in nature but an estimate of health risk due to the concentrations of air toxics measured. These estimates are applicable to the general population. They do not account for those that would be considered a sensitive population.

The uncertainties in this study primarily lie within the health risk assessment. The health‐risk method used in this study is based on an EPA method of estimating both cancerous and non‐cancerous inhalation‐based health risk. The upper value of 7.8 × 10^−6^ (μg/m^3^)^−1^ for the benzene IUR in US EPA (2021) IRIS database was used in the calculations, compared to a lower value of 2.2 × 10^−6^ (μg/m^3^)^−1^. The timelines of the wildfires sampled in this study did not fit the parameters to be considered an acute exposure or a chronic exposure, thus sub chronic exposure was chosen for the actual wildfire events. The exposure frequency, EF, of this calculation was chosen to be one month for all four wildfires, regardless of if the wildfire lasted that long. This was to establish uniformity across all fires. To predict the health risk if these fires continued in this one month pattern every year for either the occupational, residential, or lifetime scenarios were used (25, 26‐ or 70‐year respectively). These are projections for wildfire events only and may not occur at this frequency.

Although there may be limitations to this type of analysis, using measured concentrations and relating it to human health helps bridge the gaps between scientists, public health, and the general population. There have been extensive aerial studies on biomass burning composition and speciation, all of which have contributed to the important knowledge of what is emitted during wildfires. However, risk analysis involving measurements taken on the ground, where the people are and will be experiencing the smoke, seem to be lacking and even more so with relating these VOC concentrations to health risk. This study has done both, which included a large range of VOCs measured.

## 4 Conclusions

An increase in wildfire frequency and duration has caused a need for not only research of the environmental and climate impacts of wildfire smoke but also how wildfire smoke affects communities. Ground‐based wildfire smoke sampling via dual sorbent air samplers was conducted in Idaho and Washington during the 2019 and 2020 wildfire seasons. Composition of samples was determined through the standardization of 106 VOCs and subsequent analysis through TD‐GC‐MS. Aromatic, aliphatic, oxygenated and biogenic VOCs were present to various degrees in each of four fires sampled, with differences in both composition and relationships between the compounds. Quantified concentrations were used to conduct both a cancer and non‐cancer risk analysis. The main compounds found in smoke that contribute to health risk were benzene (both cancer and non‐cancer), and toluene, ethylbenzene, xylenes, and hexane for non‐cancer risk. The hazard index was well below 1 for all exposure scenarios, ranging from 0.005 to 0.007 for the wildfire event and 0.005–0.10 for the projected chronic scenarios. The cancer risk due to benzene ranged was low or 1 × 10^−6^ extra cancers per million people for the wildfire events, due to such a short exposure over a lifetime. Projected cancer risk for repeated 30‐day wildfire smoke exposure ranged from 1 to 6 × 10^−6^ for occupational exposure, 1–7 × 10^−6^ for residential scenario, and 3–19 × 10^−6^ for lifetime scenario, compared to a background risk of 1 × 10^−6^. This works adds to the limited number of health risk estimates due to wildfire pollution using ground based (exposure) measurements of gas phase organic compounds.

## Conflict of Interest

The authors declare no conflicts of interest relevant to this study.

## Supporting information

Supporting Information S1

## Data Availability

The chemical and risk data generated and used in the study are available at Mendeley Data, Johnston, N. via https://doi.org/10.17632/nchppjr9nr.1 with CC BY 4.0 License, open access, and NASA FIREX‐AQ 2019 Archive, Johnston, N. Mahluf, F, Blake, D., and Yacovitch, T directories with Earthdata registration via https://doi.org/10.5067/SUBORBITAL/FIREXAQ2019/DATA001. In addition, a supplement file for this publication is available: Dickinson et al., 021,622 Supplement. pdf Johnston, N. A. C (2021). LCSC VOC 2020 NW Fire Data set [Data set], Mendeley Data, V1, https://doi.org/10.17632/nchppjr9nr.1 Johnston, N. A. C (2022). Health Risk Data ‐ Volatile Organic Compounds in Wildfire Smoke During the 2019 FIREX‐AQ Campaign and Beyond [Data set]. Mendeley Data. V1, https://doi.org/10.17632/zgfxh8xxgm.1 NASA (2020). FIREX‐AQ 2019 Archive [Data set]. Earthdata, Blake, D., Johnston, N., Majluf, F., and Yacovitch, T. directories. https://doi.org/10.5067/SUBORBITAL/FIREXAQ2019/DATA001 Supplement File: Dickinson et al., 021,622 Supplement. pdf.
