# Supplementary material for: Health Risk Implications of Volatile Organic Compounds in Wildfire Smoke During the 2019 FIREX‐AQ Campaign and Beyond
Source: Geohealth. 2022 Aug 1;6(8):e2021GH000546. doi: 10.1029/2021GH000546 (PMC9393878; doi:10.1029/2021GH000546)
Supplement: Supplementary file 1 — Supporting Information S1 [file GH2-6-e2021GH000546-s001.pdf]

Health Risk Implications of Volatile Organic Compounds in Wildfire Smoke During the 2019 FIREX-AQ Campaign and Beyond

Gabrielle N. Dickinson, Dylan D. Miller, Aakriti Bajracharya, William Bruchard, Timbre A. Durbin, John K. P. McGarry, Elijah P. Moser, Laurel A. Nuñez, Elias J. Pukkila, Phillip S. Scott, Parke J. Sutton, Nancy A. C. Johnston

Physical, Life, Movement, and Sport Sciences Division, Lewis-Clark State College, Lewiston, ID USA

Corresponding Author: Nancy A. C. Johnston (najohnston@lcsc.edu)

Table S1: Thermal desorption-gas chromatography-mass spectrometry analysis specifications for compounds analyzed.

Figure S1: Example Carcinogenic Health-Risk Assessment Calculation

Figure S2: Photograph of Williams Flats Smoke Plume

References:

US EPA. (2016). ProUCL 5.1. Accessible at https://www.epa.gov/land-research/proucl-software .

U.S. EPA. (2021). Integrated Risk Assessment System. Retrieved October 1, 2021, from https://www.epa.gov/iris

Table S1. TD-GC-MS Specifications for Compounds Analyzed

	Compound	*Formula*	*MW (amu)*	*R.T. (min)*	*Q-ion (m/z)*	*Slope response*	R^2	*LOD (ppbv)*
1	Dichlorodifluoromethane	CCl2F2	120.91	4.131	85	1.08E+06	0.9998	0.002
2	Ethane, 1,2-dichloro-1,1,2,2-tetrafluoro	C2Cl2F4	170.92	4.371	85	3.48E+05	0.9995	0.002
3	1-Butene	C4H8	56.11	4.668	56	1.93E+05	0.9958	0.027
4	Ethene, chloro-	C4H4Cl4	193.90	4.716	62	3.84E+05	0.9992	0.003
5	2-Butene (Z), cis	C4H8	56.11	4.839	56	2.40E+05	0.9966	0.009
6	2-Butene (E), trans	C4H8	56.11	5.010	56	2.96E+05	0.9969	0.008
7	Ethyl Chloride	C2H5Cl	64.51	5.449	64	1.99E+05	0.9971	0.004
8	Isopentane	C5H12	72.11	5.595	57	2.16E+05	0.9957	0.007
9	Trichloromonofluoromethane	CCl3F	137.37	5.867	101	8.08E+05	0.9995	0.003
10	1-Pentene	C5H10	70.13	5.886	55	2.82E+05	0.9980	0.006
11	2-Pentene (Z), cis	C5H10	70.13	6.177	55	4.38E+05	0.9706	0.015
12	Isoprene	C5H8	68.12	6.303	67	2.81E+05	0.9961	0.021
13	Ethene, 1,1-dichloro-	C2H2Cl2	96.94	6.585	101	7.70E+05	0.9991	0.003
14	Butane, 2,2-dimethyl	C6H14	86.18	6.632	71	3.21E+05	0.9974	0.041
15	Dimethyl Sulfide	C2H6S	62.14	6.689	62	2.55E+05	0.9835	0.014
16	Carbon Disulfide	CS2	76.15	6.964	76	1.13E+06	0.9994	0.004
17	Methylene Chloride	CH2Cl2	84.93	7.179	84	3.81E+05	0.9998	0.003
18	Pentane, 2-methyl	C6H14	86.18	7.236	71	2.88E+05	0.9989	0.040
19	Ethylene, 1,2-dichloro (E), trans	C2H2Cl2	96.94	7.558	61	4.32E+05	0.9998	0.002
20	Pentane, 3-methyl	C6H14	86.18	7.565	57	3.91E+05	0.9983	0.035
21	n-Hexane	C6H14	86.18	7.890	57	4.33E+05	1.0000	0.007
22	Ethane, 1,1-dichloro-	C2H4Cl2	98.96	8.061	63	6.10E+05	0.9989	0.003
23	1-Hexene	C6H12	84.16	8.535	56	2.21E+05	0.9990	0.011
24	Pentane, 2,4-dimethyl	C7H16	100.20	8.548	57	2.53E+05	0.9935	0.067
25	Cyclopentane, methyl-	C6H12	84.16	8.700	56	5.82E+05	0.9995	0.017
26	Ethylene, 1,2-dichloro (Z), cis	C2H2Cl2	96.94	8.760	61	6.71E+05	0.9920	0.003
27	Trichloromethane	CHCl3	119.38	9.105	83	6.73E+05	0.9996	0.002
28	Hexane, 2-methyl-	C7H16	100.20	9.370	85	2.95E+05	0.9996	0.031
29	Ethane, 1,1,1-trichloro-	C2H3Cl3	133.40	9.408	97	4.06E+05	0.9998	0.006
30	Cyclohexane	C6H12	84.16	9.531	56	4.73E+05	0.9999	0.009
31	Hexane, 3-methyl	C7H16	100.20	9.588	71	2.79E+05	0.9996	0.035
32	Carbon Tetrachloride	CCl4	153.82	9.633	117	1.60E+05	0.9991	0.010
33	Ethane, 1,2-dichloro-	C2H4Cl2	98.96	9.832	62	1.03E+06	0.9961	0.002
34	Benzene	C6H6	78.05	9.860	78	9.74E+05	0.9993	0.014
35	Heptane	C7H16	100.20	10.101	71	3.32E+05	0.9987	0.006
36	Trichloroethylene	C2HCl3	131.40	10.629	130	5.62E+05	0.9994	0.004
37	Propane, 1,2-dichloro-	C3H6Cl2	112.98	10.885	63	3.30E+05	0.9992	0.005
38	Cyclohexane, methyl	C7H14	98.19	10.932	83	4.57E+05	0.9968	0.039
39	1,4-Dioxane	C4H8O2	88.11	11.008	88	2.81E+05	0.9995	0.006
40	Methane, bromodichloro-	CHBrCl2	163.8	11.188	83	3.64E+05	0.9983	0.004
41	Pentane, 2,3,4-trimethyl	C8H18	114.23	11.388	71	3.69E+05	0.9981	0.045

	Compound	*Formula*	*MW (amu)*	*R.T. (min)*	*Q-ion (m/z)*	*Slope response*	R^2	*LOD (ppbv)*
42	Heptane, 2-methyl	C8H18	114.23	11.574	57	5.28E+05	0.9987	0.027
43	Ethane, 1-bromo-2-chloro-	C2H4BrCl	143.41	11.647	63	3.50E+04	0.9942	NA
44	1-Propene, 1,3-dichloro-	C3H4Cl2	110.97	11.754	75	6.12E+05	0.9991	0.005
45	Heptane, 3-methyl	C8H18	114.23	11.770	85	3.99E+05	0.9989	0.033
46	Methyl Isobutyl Ketone	C6H12O	100.16	11.878	58	2.94E+05	0.9995	0.005
47	Dimethyl Disulfide	C2H6S2	94.20	11.928	94	8.04E+05	0.9976	0.004
48	Toluene	C7H8	92.14	12.254	91	1.34E+06	0.9985	0.005
49	Octane	C8H18	114.26	12.314	85	4.23E+05	0.9979	0.030
50	1-Propene, 2,3-dichloro-	C3H4Cl2	110.97	12.444	75	5.67E+05	0.9993	0.004
51	Ethane, 1,1,2-trichloro-	C2H3Cl3	133.40	12.712	97	4.96E+05	0.9996	0.005
52	2-Hexanone	C6H12O	100.16	12.975	58	3.77E+05	0.9993	0.006
53	Tetrachloroethylene	C2Cl4	165.83	13.032	166	7.99E+05	0.9988	0.005
54	Methane, dibromochloro-	CHBr2Cl	208.28	13.301	129	8.80E+05	0.9992	0.005
55	Ethane, 1,2-dibromo-	C2H4Br2	187.86	13.509	107	7.49E+05	0.9990	0.006
56	Benzene, chloro-	C6H5Cl	112.56	14.189	112	1.15E+06	0.9979	0.007
57	Nonane	C9H20	128.25	14.404	57	4.94E+05	0.9877	0.041
58	Ethylbenzene	C8H10	106.17	14.458	91	1.49E+06	0.9973	0.006
59	Xylene (m,p)	C8H10	106.16	14.458	91	9.56E+05	0.9869	0.078
60	Xylene (o)	C8H10	106.16	15.043	91	9.54E+05	0.9846	0.038
61	Styrene	C8H8	104.06	15.046	104	8.08E+05	0.9842	0.007
62	Methane, tribromo-	CHBr3	252.73	15.343	173	9.29E+05	0.9981	0.006
63	Benzene, (1-methylethyl)	C9H12	120.19	15.555	105	1.41E+06	0.9860	0.040
64	α-Pinene	C10H16	136.23	15.590	93	1.21E+05	0.9938	0.016
65	Ethane, 1,1,2,2-tetrachloro-	C2H2Cl4	167.85	15.903	83	8.42E+05	0.9966	0.010
66	Benzene, propyl-	C9H12	120.20	16.156	91	1.54E+06	0.9810	0.048
67	Benzene, 1-ethyl-3-methyl	C9H12	120.19	16.270	105	1.26E+06	0.9861	0.063
68	Benzene, 1-ethyl-4-methyl-	C9H12	120.19	16.311	105	1.19E+06	0.9828	0.011
69	Decane	C10H22	142.28	16.327	57	4.79E+05	0.9831	0.061
70	Mesitylene	C9H12	120.19	16.390	105	1.01E+06	0.9801	0.051
71	Sabinene	C10H16	136.23	16.402	93	2.41E+05	0.9951	0.020
72	β-Pinene	C10H16	136.23	16.576	93	1.06E+05	0.9974	0.032
73	Benzene, 1-ethyl-2-methyl	C9H12	120.19	16.965	105	1.31E+06	0.9828	0.053
74	Benzene, 1,2,4-trimethyl-	C9H12	120.19	16.965	105	5.51E+05	0.9851	0.072
75	D-Limonene	C10H16	136.23	17.373	68	1.36E+05	0.9959	0.031
76	Benzene, 1,3-dichloro-	C6H4Cl2	147.00	17.440	146	1.02E+06	0.9933	0.018
77	Benzene, 1,2-dichloro-	C6H4Cl2	147.00	17.563	146	1.02E+06	0.9930	0.021
78	Benzene, 1,2,3-trimethyl	C9H12	120.19	17.617	105	2.66E+06	0.9750	0.046
79	Benzyl Chloride	C7H7Cl	126.58	17.727	91	1.12E+06	0.9940	0.013
80	Phenol, 2-chloro-	C6H5ClO	128.55	17.727	128	2.89E+05	0.9992	NA
81	γ-Terpinene	C10H16	136.23	17.847	93	2.23E+05	0.9925	0.029
82	Benzene, 1,3-diethyl	C10H14	134.22	17.863	119	8.68E+05	0.9931	0.044
83	Benzene, 1,4-diethyl	C10H14	134.22	17.863	119	8.79E+05	0.9923	0.048
84	Phenol	C6H6O	94.11	17.933	94	2.85E+05	0.9987	0.094

	Compound	*Formula*	*MW (amu)*	*R.T. (min)*	*Q-ion (m/z)*	*Slope response*	R^2	*LOD (ppbv)*
85	Undecane	C11H24	156.31	18.110	57	6.24E+05	0.9965	0.078
86	Benzene, 1,4-dichloro-	C6H4Cl2	147.00	18.161	146	9.07E+05	0.9922	0.017
87	Terpinolene-a	C10H16	136.23	18.448	121	1.32E+05	0.9981	0.023
88	Sabinene hydrate	C10H18O	154.25	18.685	93	3.88E+04	0.9905	0.086
89	Guaiacol	C7H8O2	124.14	19.052	109	2.02E+05	0.9894	0.039
90	L-Fenchone	C10H16O	152.23	19.330	81	2.68E+05	0.9962	0.018
91	Fenchol	C10H18O	154.25	19.919	81	1.31E+05	0.9978	0.021
92	Dodecane	C12H26	170.33	20.039	57	7.22E+05	0.9929	0.055
93	Phenol, 2-nitro-	C6H5NO3	139.11	20.636	139	1.32E+05	0.9984	NA
94	Camphor	C10H16O	152.23	20.921	95	2.38E+05	0.9955	0.016
95	Phenol, 2,4-dimethyl-	C8H10O	122.16	21.038	122	2.29E+05	0.9992	NA
96	Benzene, 1,2,4-trichloro-	C6H3Cl3	181.45	21.180	180	1.06E+06	0.9805	0.059
97	Borneol	C10H18O	154.25	21.297	95	5.10E+05	0.9955	0.022
98	α-Terpineol	C10H18O	154.25	21.455	136	6.76E+04	0.9986	NA
99	1,3-Butadiene, 1,1,2,3,4,4-hexachloro-	C4Cl6	260.76	21.557	225	5.00E+05	0.9868	0.068
100	Phenol, 2,4-dichloro-	C6H4Cl2O	163.00	22.012	162	2.16E+05	0.9992	NA
101	Geraniol	C10H18O	154.25	22.638	69	1.40E+05	0.9963	0.051
102	Pulegone	C10H16O	152.23	23.043	152	8.51E+04	0.9999	0.021
103	Phenol, 4-chloro-3-methyl-	C7H7ClO	142.58	26.761	107	1.90E+05	0.9979	NA
104	Syringol	C8H10O3	154.16	27.235	154	1.20E+05	0.9847	0.013
105	Phenol, 2,4,6-trichloro-	C6H3Cl3O	197.40	28.206	196	1.45E+05	0.9983	NA
106	α-Cedrene	C15H24	204.35	28.747	119	2.26E+05	0.9993	0.013

Figure S1. Example Carcinogenic Health-Risk Assessment Calculation

The exposure concentration (EC) was calculated from benzene concentrations found in air samples collected at each fire. The upper confidence level (UCL) determined by the EPA ProUCL 5.1 (2016) software equals the contaminant concentration in air (CA) in the sub chronic inhalation risk calculation:

$$EC = \sum \frac{CA \times ET \times EF \times ED}{AT}$$

This example will cover benzene in the Nethker Fire for the *residential scenario only*.

The assumptions are the levels of benzene reoccur for 1 month of every year for 26 years.

All concentration units in this calculation are μg/m^3.

CA (Nethker Fire) = $\dfrac{8.26\ ppbv \times 78\ \frac{g}{mole}}{24.45\ \frac{L}{mole}} = 26.4$ μg/m^3

CA (background McCall, ID; nearest passive site) = $\dfrac{0.081\ ppbv\ x\ 78\ \frac{g}{mole}}{24.45\ \frac{L}{mole}}$ = 0.258 μg/m^3

Exposure time (ET) = 24 hours, fire was occurring 24 hours per day.

Exposure frequency (EF)= 30 days (fire event) and 320 days (background).

Exposure duration (ED) = 26 years (residential scenario).

Averaging time (AT) is the same regardless of which scenario = (24 hours x 365 days x 70 years).

EC = $\dfrac{26.4\frac{ug}{m^3}\times 24\frac{hrs}{day}\times 30\ days \times 26\ years}{24\frac{hrs}{day}\times 365\ days \times 70\ years} + \dfrac{0.258\frac{ug}{m^3}\times 24\frac{hrs}{day}\times 320\ days \times 26\ years}{24\frac{hrs}{day}\times 365\ days \times 70\ years}$ = **0.886 μg/m^3**

The summation accounts for both concentrations of the contaminant during the fire event, as well as the background concentrations in that area during the other times of the year.

This value is then used to calculate the cancer risk associated with inhalation of a carcinogenic compound. The equation is as follows:

Risk = IUR x **EC**

The inhalation unit risk (IUR) for benzene is 7.8 x 10^{-6} (μg/m^3)$^{-1}$ (US EPA, 2021). The risk associated with the benzene concentrations found in the Nethker Fire was:

Risk = 7.8 x 10^{-6} (μg/m^3)$^{-1}$ x 0.886 μg/m^3 = 1.9 x 10^{-5}

This value is then scaled to one million because this is the EPA reference population metric for discussing health risk. Thus, the health risk is 19 x 10^{-6}.

Therefore, the health risk from the concentrations of benzene found in the Nethker Fire ended up having the potential to cause ***19 extra cancers per million people.***

Figure S2. Photograph of Williams Flats Smoke Plume

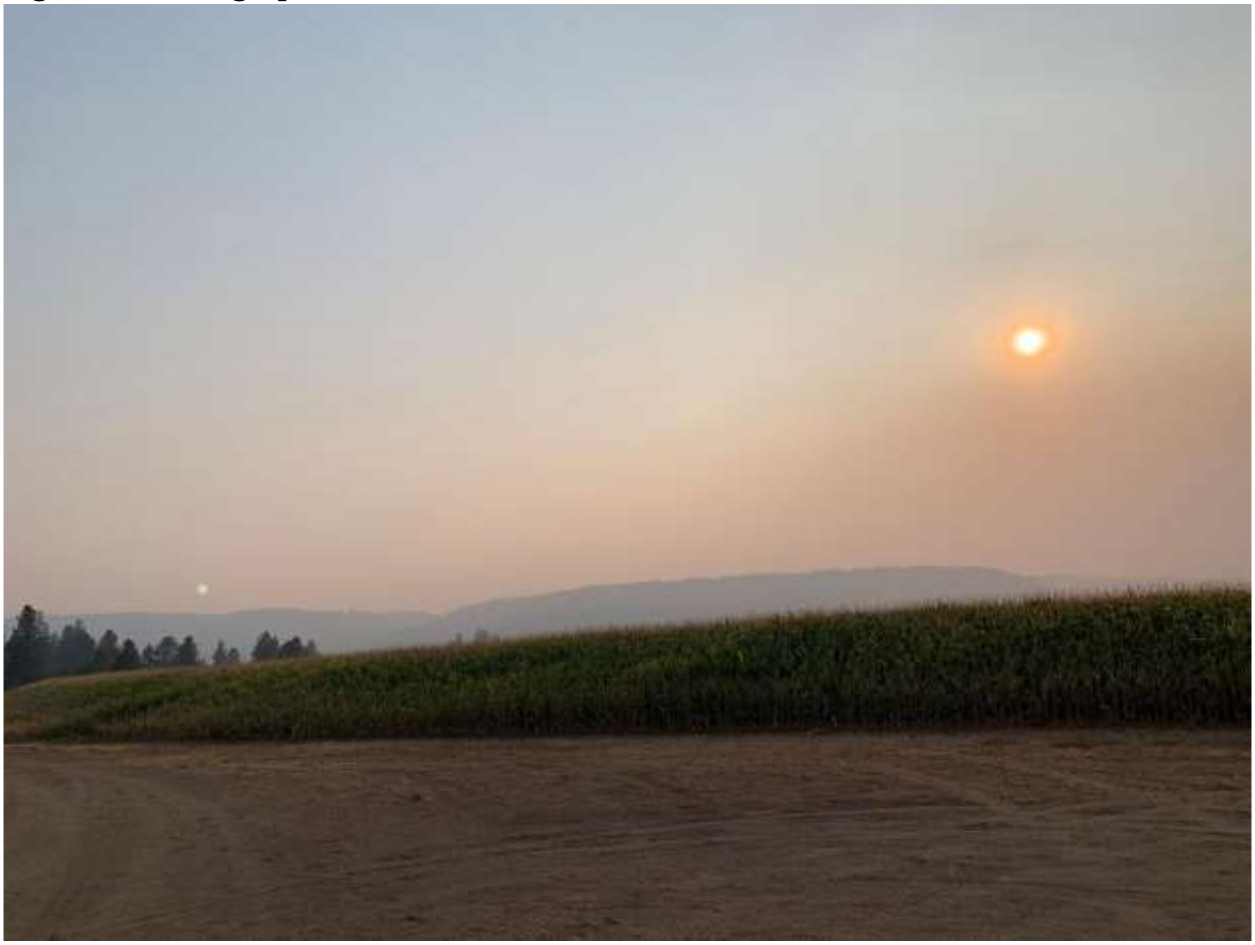

Photograph of 3 August 2019 sampling about 30 miles east of Williams Flats Fire, Eastern Washington.

Photograph taken by: Dr. Nancy A. C. Johnston
